# Gender and Contraception in Kenya: a Scoping Review

**DOI:** 10.1186/s12978-026-02443-5

**Published:** 2026-09-24

**Authors:** Simran Suri, Sarah Milton

**Affiliations:** King’s College London, London, UK

**Keywords:** Contraception, Sex, Kenya, Gender, Decision-making, Relationships, Education, Peers, Stigma, Childbearing.

## Abstract

**Background:**

Access to the full range of contraceptive methods is a core component of sexual and reproductive health. While Kenya had one of the highest rates of contraceptive use compared to other countries on the African continent, studies since 2015 have shown prevalence of use has fallen; contraceptive usage rates are estimated at 38.7%, significantly lower than Kenya’s target of 62% and in a context of high rates of unsafe abortions and high maternal mortality. This scoping review investigates the role of gender norms in determining patterns of contraceptive usage among women of childbearing age in Kenya.

**Methods:**

A PRISMA-ScR approach was employed, involving a keyword search of population, concept and context using ProQuest. The 307 search results were iteratively screened using specific eligibility criteria, and rigorous JBI checklist critical appraisal was conducted. Data relevant to the aim was extracted, synthesised and distilled, ensuring comprehensive, rigorous and reproducible scoping of a final 16 papers.

**Results:**

Studies showed that heteronormative and gendered norms influenced contraceptive use, unmet need and discontinuation, at social, societal and structural levels. Heterosexual male partner preferences largely shaped women’s contraceptive decisions, with disapproval or intimate/gender-based violence triggering covert usage facilitated by injectable methods. Networks, involving friends, kin and healthcare providers modulated perceived gender norms, and therefore contraceptive decisions. Stigmatisation against adolescents’ and premarital sexuality was identified as an important barrier to contraceptive use, as well as perceived marital duties of coitus and childbearing. As expected, female education, employment and wealth were structural determinants shaping access and understanding of contraception. The evidence base was largely concentrated in Western Kenya.

**Conclusions:**

This review presents a comprehensive account of the influence of gender norms on contraceptive usage patterns, though addressing sociocultural norms is complex. Public health interventions can improve contraceptive counselling by providers, involve males due to their decision-making dominance, and improve service accessibility by integrating HIV, contraceptive and domestic violence services. Wider structural factors surrounding education, employment and subsequent socioeconomic mobility for women will always shape the success of interventions. Further research could identify how this varies by region or demographic.

**Plain English Summary:**

Women’s access to contraception is an important health issue. Kenya aims for 62% of reproductive-age women to use contraception and had high rates of contraceptive use compared to other African countries. However, since 2015, contraceptive use fell well below that target, while rates of unsafe abortions and maternal deaths remain high. This review summarises women’s beliefs about what society expects and considers acceptable, and their impact on contraceptive use, particularly within Western Kenya.

Women’s decisions about contraception are affected by the opinions of their partners and others in their social circle (such as friends, family, and healthcare providers). In heterosexual romantic relationships, studies showed that in this context male partners often have more power than women, so their disapproval can cause women to avoid contraception or use it secretly. When people in a woman’s social circle have disapproving or negative opinions about contraception, this can have a similar effect. Studies showed that both reproducing and satisfying male intimate partners were conceptualised as important, gendered duties, leading to the avoidance of contraception. Levels of education, financial security and employment led to greater decision-making agency in most studies, associated with greater access to information about contraception including from peers.

Involving men in family planning discussions, training healthcare providers to discuss contraception non-judgementally and combining contraceptive services with other healthcare services to improve access could lead to increased contraceptive use.

**Supplementary Information:**

The online version contains supplementary material available at https://doi.org/10.1186/s12978-026-02443-5.

## Background

Access to the full range of contraceptive methods is a core component of sexual and reproductive health [1]. Modern contraception refers to products or surgical procedures that interfere with reproduction resultant from heterosexual intercourse [2], including intrauterine devices, subdermal implants, oral contraceptive pills, injectables, male/female condoms, patches, vaginal rings, female sterilisation [3] and male sterilisation. Until 2015 when prevalence of use stalled, Kenya had one of the highest rates of contraceptive use compared to other countries on the African continent [4]. Studies now estimate a contraceptive usage rate of 38.7%, significantly lower than Kenya’s target of 62% [5]. Suggested explanations for this phenomenon include reduced availability of contraception, diversion of resources to HIV/AIDS care, insufficient support for family planning programmes, a lack of positive male involvement in family-planning, contraceptive misconceptions including fears of medical or physical complications, and cultural perceptions surrounding the acceptability of contraceptive use among different groups [5].

Below-target contraceptive usage rates are an important issue. Kenya’s maternal mortality increased by 55% between 2017 and 2020, ranking among the highest globally [6]. While multifactorial, high maternal deaths can be partially explained by low contraceptive uptake leading to unsafe termination of unwanted pregnancies which could have been prevented by contraceptive use. Anti-abortion laws in Kenya result in informal and poorly regulated abortions, which are associated with high fatalities [7]. Improving contraceptive uptake would provide a safer alternative to informal abortions as a means of preventing unwanted pregnancies, with a systematic review suggesting that improving contraceptive use across developing regions globally could reduce maternal deaths by up to 73% [8].

Contraceptive decision-making is closely related to gender-based phenomena, as evidenced by data from the Kenyan DHS [9]. 42% of married, heterosexual women believe that contraceptive use is mainly the wife’s decision, 50% believe it to be a joint decision between spouses, and 7% believe that it is a male decision; rates of participation in decisions are 18% higher amongst married women who have completed secondary education than those who have not. Additionally, 11% of married, heterosexual women report being pressured by their partners or other family member to become pregnant when they did not want to. The former statistic implies that there may exist a relationship between education level and negotiation power and agency in relationships, while the latter alludes to gender-based expectations surrounding the female role of childbearing.

Gender norms are definable as shared social rules about how women and men are expected to behave in a society, which play a role in shaping individuals’ access to resources, opportunities and behaviours [10]. To understand contraceptive practices, one must situate Kenyan gender norms within overarching theories of power and social influence. Kabeer, a prominent scholar in gender and development, argues that women’s empowerment comprises three interrelated components: resources, agency and achievements [11]. In terms of contraceptive practices, material and social resources can include access and engagement with contraceptive services, financial means, education, freedom from stigma and the absence of spousal or peer resistance. Agency, consisting of autonomy, decision-making and negotiation power within intimate relationships, translates ‘resources’ into ‘achievements’; with achievements defined as the ability to realise one’s own reproductive goals, such as contraceptive uptake and sexual self-determination. These processes may be shaped by gender norms governing female empowerment, decision-making agency, and reproductive roles, that will be discussed in this review.

While studies tend to document the determinants of Kenyan contraceptive usage by region, there is limited national synthesis of this evidence, from which reproductive health policies and service development could benefit with regard to the development of policy and programmes that reflect population needs. This scoping review therefore aims to investigate the mediators of the gender-contraception relationship in Kenya, asking ‘what is the role of perceived gender norms in determining patterns of contraceptive usage among women of childbearing age in Kenya?’.

## Methodology

The scoping review followed PRISMA-ScR guidelines [12], to perform a critical synthesis of the literature in an iterative and inductive manner [13]. The review’s aim was directed by the Population, Concept, Context (PCC) framework [14], whereby the population was women of childbearing age, in the context of those living in Kenya, and relating to the concept of gendered contraceptive use.

### Selection criteria

Following the PCC framework, the eligibility criteria are presented in Table 1, consisting of detailed, specific inclusion and exclusion criteria relevant to the aim of this review.

**Table 1:** Inclusion criteria mapped to population, concept and context

PCC	Eligibility criteria
Population	To include Kenyan women of childbearing age (15–49 years), spouses/male partners, other key social influences (peers, family, healthcare providers). Transgender and non-binary experiences are important research avenues, but are outside the scope of this review.
Concept	To include modern contraceptives (intrauterine devices, pills, injectables, implants, patches, intravaginal rings, sterilisation, condoms (when used for pregnancy prevention only)). Literature discussing gender roles, expectations, beliefs and autonomies of Kenyan women (and their social networks) surrounding sexual activity and contraceptive usage. Qualitative, quantitative, mixed-methods studies and other reviews. Emergency contraception may be associated with different priorities and beliefs to regular contraception and is therefore beyond the scope of this review.
Context	To include national/regional accounts of Kenyan findings, with multi-regional inclusion (to avoid inaccurate homogenisation of perceptions across regions). To exclude: literature with focus outside Kenya.

### Search strategy

Due to the multidisciplinary nature of social science questions [15], ProQuest, a comprehensive platform, was selected to encapsulate both biomedical and social science based sources across a broad scope of 47 databases, with an initial search phrase (‘impact of perceived gender norms on modern contraceptive use in Kenya’) yielding 6,067 results A concept map of keywords relating to the PCC variables was created (Additional File 1), including variants/synonyms, to be separated by Boolean operators ‘AND’ and ‘OR’ in the final keyword string (Additional File 2). During screening, no additional keywords were identified. The implementation of concept mapping and an iterative search strategy were intended to maximise sensitivity within ProQuest. To encapsulate reliable, complete and recent literature, the filters ‘Full Text’, ‘Peer-reviewed’, ‘Last 10 Years’ and ‘English’ were applied. While it is acknowledged that the latter may have led to unintentional exclusion of insights written in other languages, it was felt important to mitigate against translation error that could lead to misrepresentation of findings.

### Selection of evidence sources

**Fig. 1:**
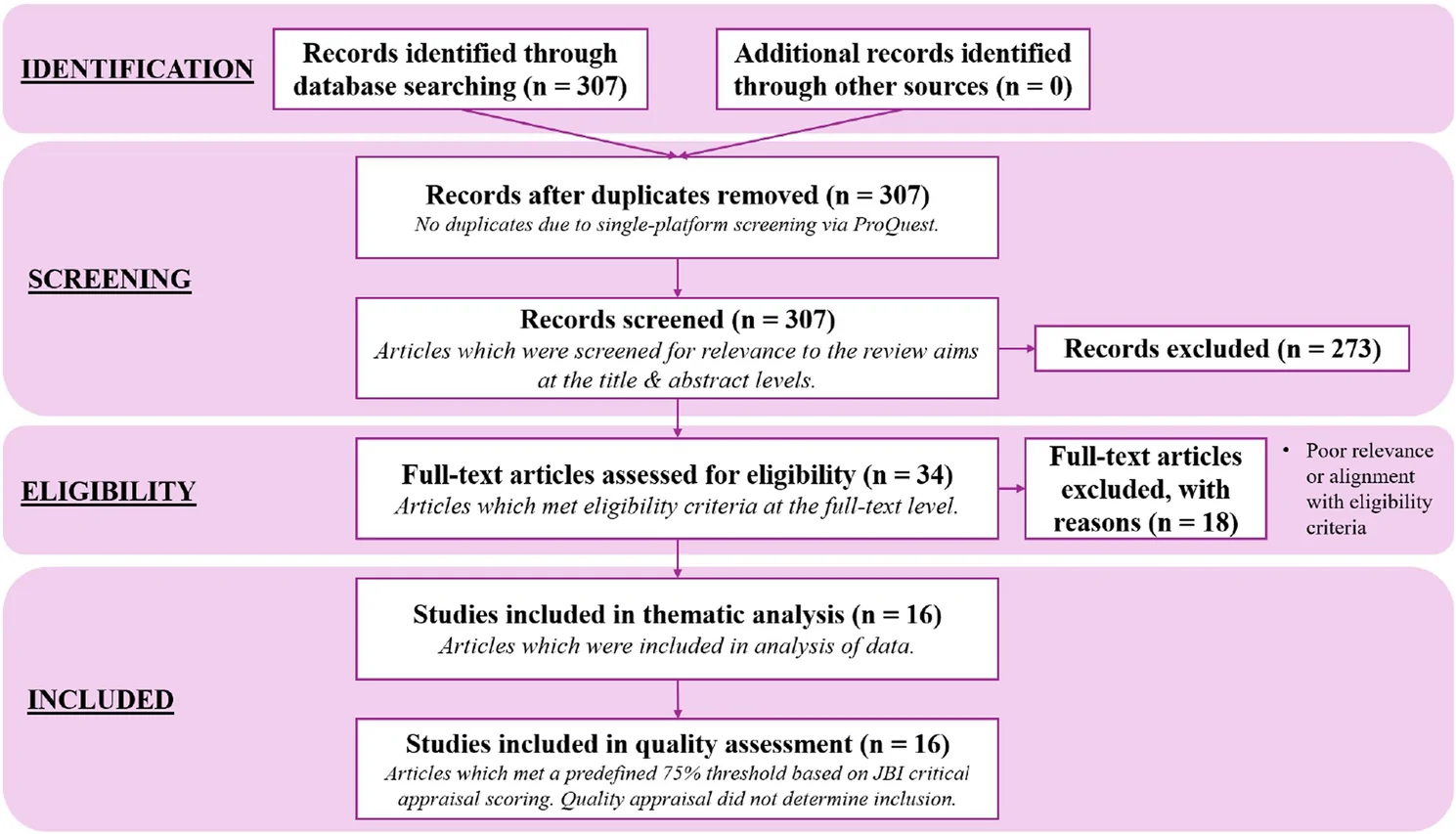
Summary of selection of evidence

Stepwise screening of studies yielded by the search terms was conducted (Fig. 1). Studies were evaluated for their relevance to the review aim, first at the title, then the abstract and then at the full-text level, excluding poorly relevant studies at each stage. Relevance was defined as studies that met the predefined inclusion and exclusion criteria. Screening and selection were conducted by the first author due to constraints during the initial phase of the project, such that no inter-author disagreement requiring consensus occurred. Formulating a priori eligibility criteria helped to minimise the possibility of selection bias. Finally, remaining studies were critically appraised against checklists from the Joanna Briggs Institute (JBI) [14] to contextualise the methodological quality of the evidence base and identify common reporting limitations to inform interpretation of findings in relation to the research question. In the checklist, ‘Yes’ items were scored 1, ‘No’ items were scored 0, and ‘Unclear’ items were scored 0.5. This approach avoided unfairly downgrading studies where the item may have been attempted but inadequately reported, leading to insufficient information to determine whether the quality control measure was implemented. It therefore reflects nuances in study reporting while still ensuring a distinction between studies with differences in which methodological limitations were present. While this approach carries a risk of overstating quality, relative scoring remains valid due to its uniform application across studies. Binary scores (Unclear = 0) are also available in the supplementary materials for reference. Fractional scores were then converted to percentages to homogenise scores across checklists with different numbers of ‘applicable’ checkpoints. A percentage score threshold of 75% was applied to provide a descriptive summary of methodological quality across studies. Several quantitative studies possessed the same deficits surrounding identifying and mitigating confounding variables, while several qualitative studies did not adequately discuss reflexivity. In practice, no studies were excluded on the basis of the critical appraisal threshold, and all included studies met or exceeded this value. As displayed in Figs. 1 and 38 articles with relevant titles were identified, but of these, 16 were relevant at the full-text level. The process from developing keyword search terms to completing critical appraisal spanned approximately four weeks.

### Data extraction and charting

Data were extracted and organised using a charting approach to map key information and summarise findings across the included studies. This facilitated identification of patterns, allowing the data to be later considered through a theoretical lens. Characteristics for extraction were selected according to the aim, eligibility criteria and concepts relevant to PCC or derived from background research. These categories are summarised in Table 2; the left column identifies important contextual factors of the studies, while the right column categorises the key findings related to contraceptive decision-making. The full data-extraction table can be found in Additional File 3.

**Table 2:** Summary of data items extracted and charted

Study context and characteristics extracted	Key findings extracted and categorised
Title, year published and authors	Motivations/barriers for use that pertain to gender roles (miscellaneous)
Location of focus & location characteristics	Motivations for discontinuation that pertain to gender roles (miscellaneous)
Population of focus & participant inclusion criteria	Education level & accuracy of understanding of contraceptives
Contraceptive methods evaluated	Female empowerment, careers & independence
Study duration	Level of female autonomy & male spousal/ partner effects
Research aim/research gap filled	Social network influences
Data collection methods	Marital roles of childbearing and fulfilment of male pleasure
Sample size & participant sociodemographic characteristics	Stigmatisation of female sexual pleasure & contraceptive usage
	Study limitations & policy recommendations

### Data synthesis

Data synthesis involved thematic analysis of findings extracted from the included studies. Relevant findings were extracted into a structured table using the above analytical categories that reflected recurring concepts. These categories were then collated into broader themes relating to male partner effects, social network influences, stigmatisation of contraceptive use and sexual activity, perceived marital roles relating to childbearing and male pleasure, and female empowerment. Findings within each theme were synthesised into a cohesive narrative to highlight areas of convergence and variation across studies. Heterogeneity and contradictory findings were also reported. Following this synthesis, Kabeer’s ‘Resources, Agency, and Achievements’ framework was applied as an interpretive lens in the Discussion.

## Results

### Selection of evidence

The search strategy yielded 307 relevant titles, refined to 16 articles for inclusion following screening, whose characteristics are summarised in Additional File 4. Common reasons for exclusion at the full-text level included where findings were poorly gender norms-focused, did not draw significant links with contraceptive usage, or did not adequately explore sociocultural factors that may influence gender norms.

### Critical appraisal

A score threshold of 75% was selected to pragmatically summarise methodological and reporting quality; certain omissions were common across several papers that were otherwise highly valuable. In particular, many quantitative studies did not explicitly state how confounders were identified and managed, and qualitative studies often did not discuss reflexivity and positionality. All papers relevant at the full-text level received a score of at least 75% using the aforementioned fractional scoring approach, such that the final 16 papers included in the analysis were all above the applied threshold per the fractional scoring approach. However, it is acknowledged that one paper fell below this threshold per its binary score, scoring 64%.

### Characteristics of evidence

**Fig. 2:**
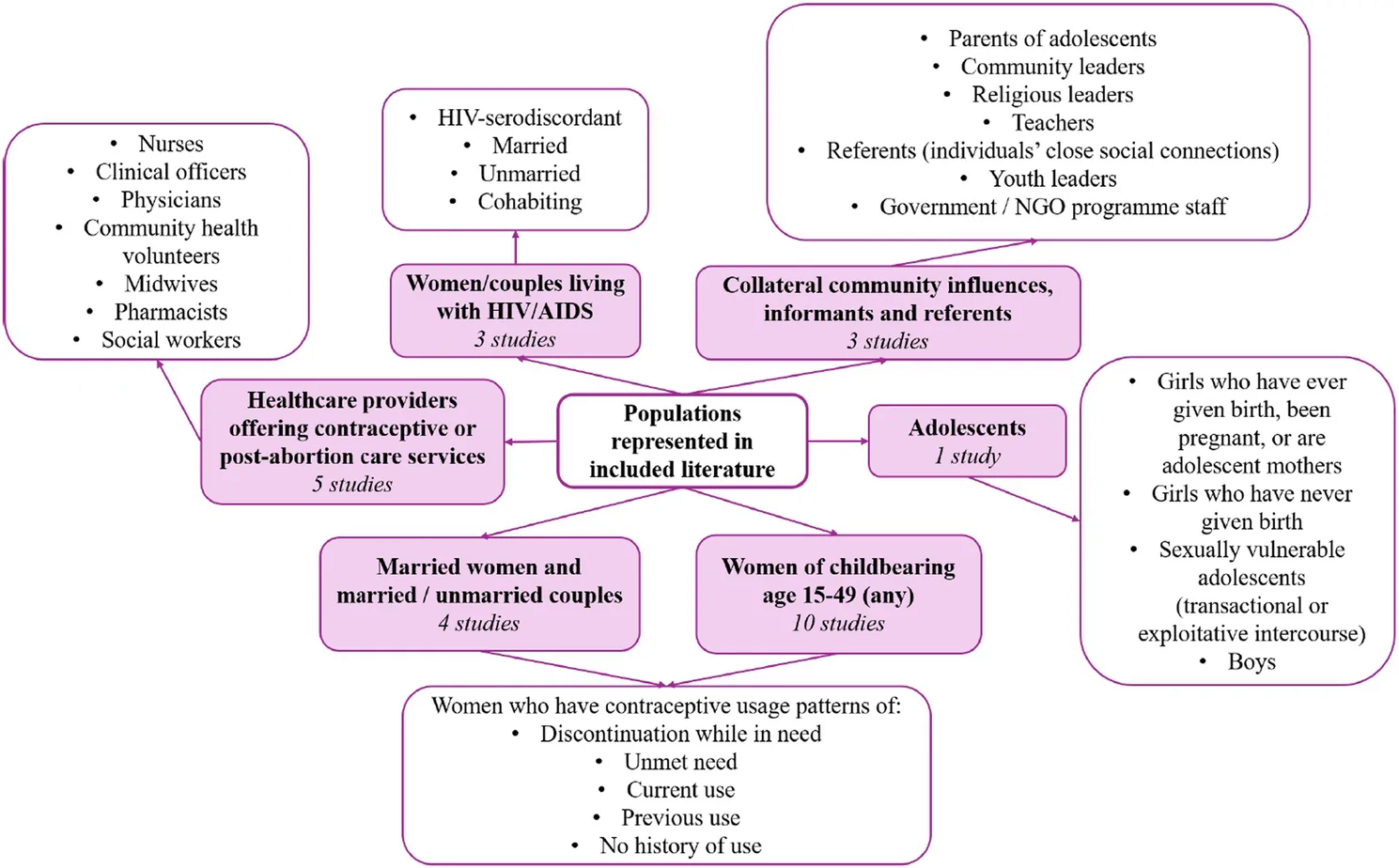
Summary of populations represented in the included literature

Literature scoping yielded data in a variety of populations and areas across Kenya. Figure 2 depicts the populations represented, alongside their prevalence within the included studies; women of childbearing age as a non-specific population were the focus of the majority of the studies, while adolescents and various social referents were explicitly represented in the minority of the data. Table 3 summarises characteristics of the included studies; there was a broad range of methods for analysing data, and both quantitative and qualitative studies were captured.

**Table 3:** Characteristics of evidence

Characteristic	Details
Study design and methods of data analysis	• Quantitative: 8/16 studies (Questionnaires, Bi/Multivariate Analysis, Logistic Regression, Latent Class Analysis, Stigma Indices, Normative Influence Modelling) • Qualitative: 4/16 studies (Focus Group Discussions, Interviews – in-depth, semi-structured, key informant interviews) • Mixed methods approach: 4/16 studies
Populations represented	• Adolescents: 6/16 studies • HIV-positive women/couples: 2/16 studies • Male spouses: 2/16 studies • Couples: 2/16 studies • Healthcare professionals: 4/16 studies • Women of childbearing age overall: 16/16 studies
Sociodemographic characteristics	Demonstrated variability of a similar level across studies.
Study location scope	• Small number of villages: 1/16 studies • Large regional scope: 11/16 studies • National scope: 4/16 studies • Studies where Western Kenya comprises at least 50% of included locations : 7/11 regional studies
Relevant concepts discussed	• Resources as structural determinants – female empowerment through education, wealth, or employment: 8/16 studies • Agency shaped by relational decision-making – male partner effect: 14/16 studies • Agency shaped by fertility and marital expectations – childbearing or provision of spousal pleasure:12/16 studies • Agency shaped by stigmatisation and social proof – social network perceptions: 6/16 studies • Agency shaped by stigmatisation and social proof – stigmatisation of sexual activity and contraceptive use: 7/16 studies

**Fig. 3:**
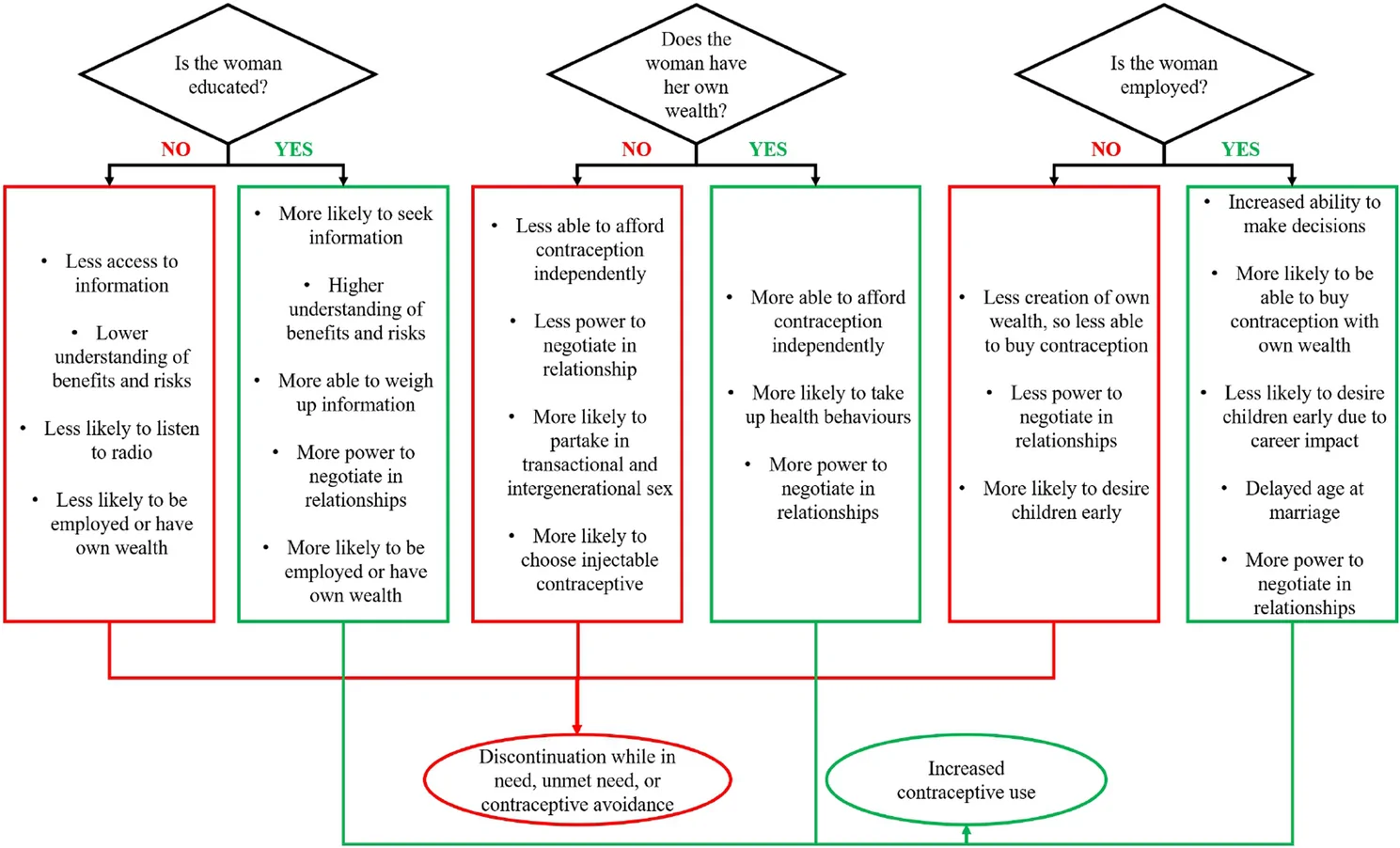
Key findings related to levels of female independence and empowerment

**Fig. 4:**
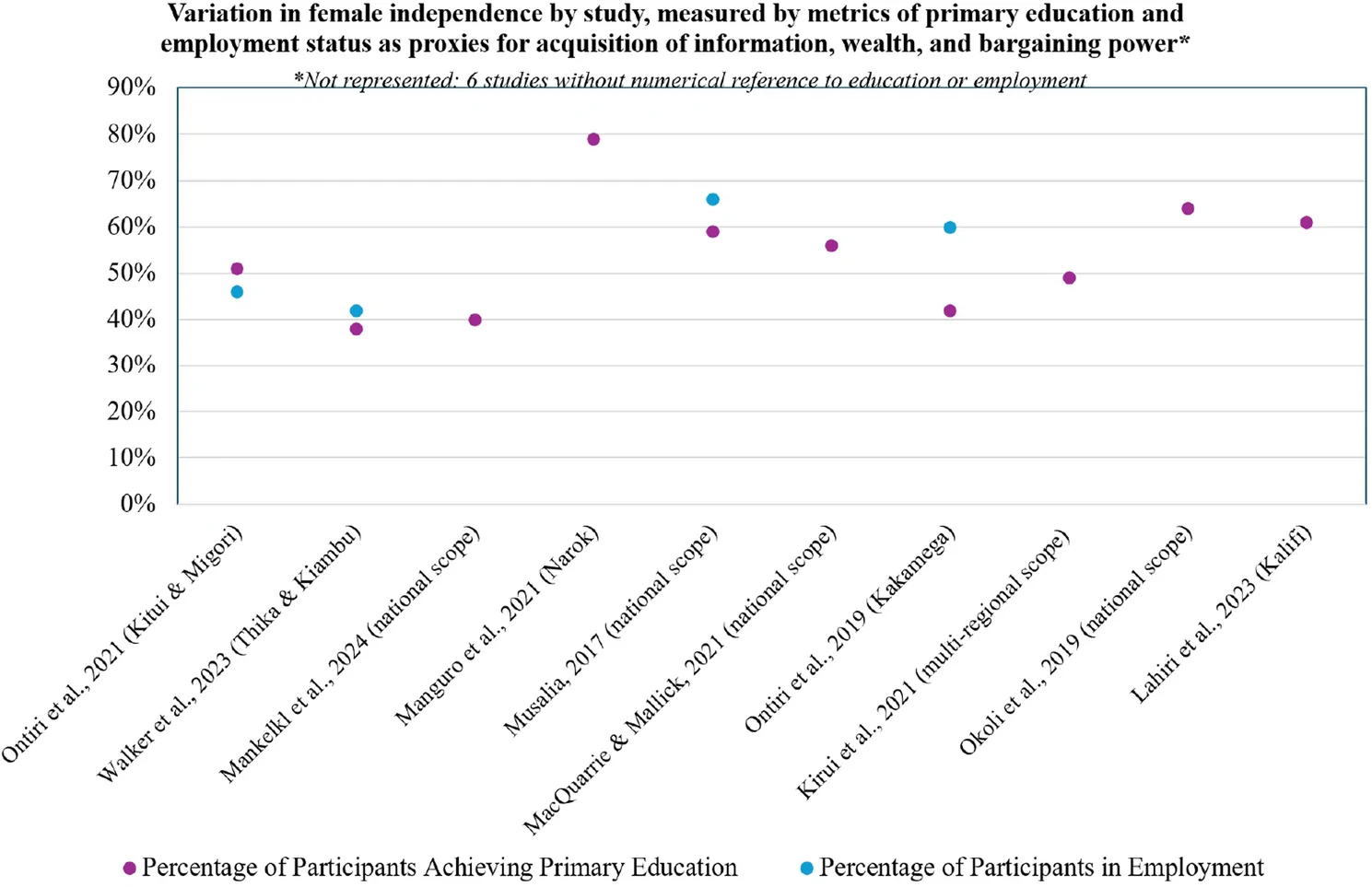
Variation in education and employment status across included studies

**Fig. 5:**
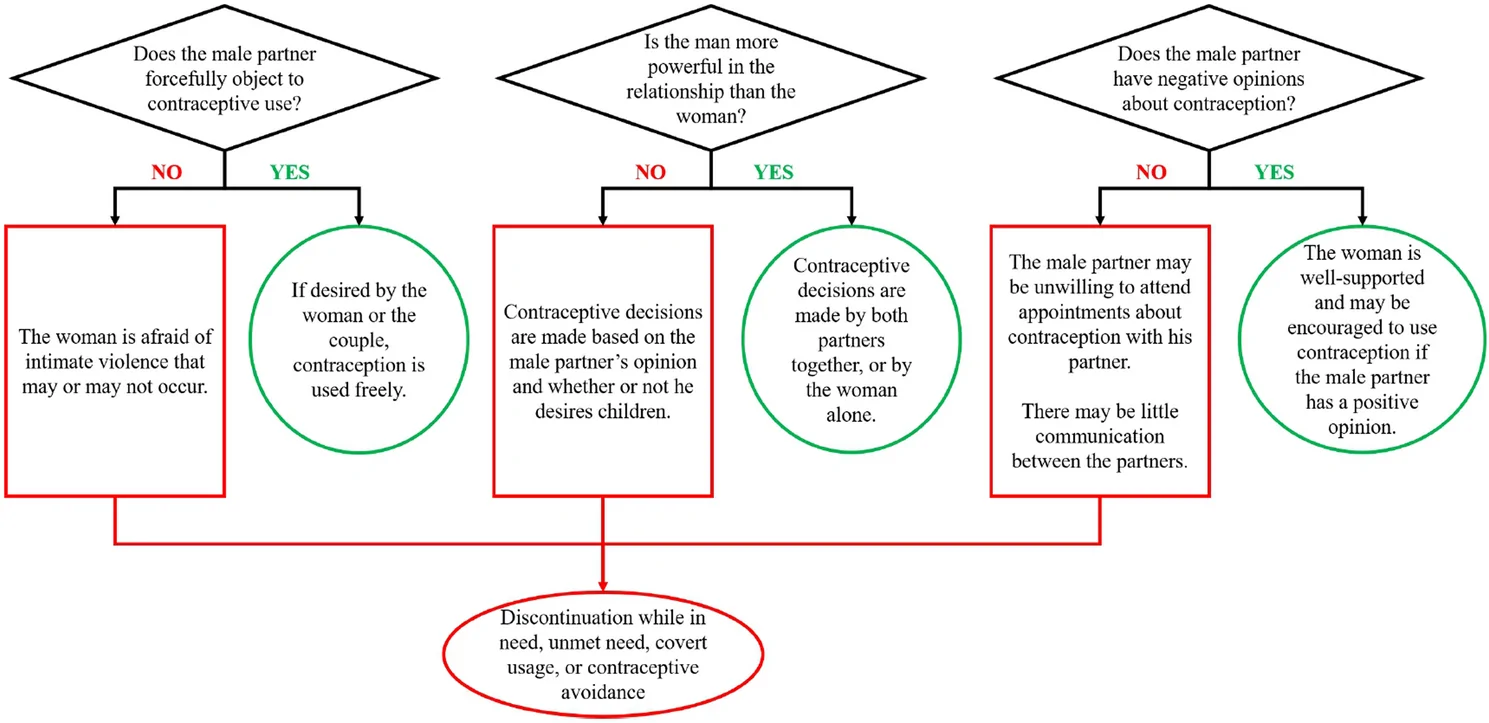
Key findings related to spousal/male partner effects

**Fig. 6:**
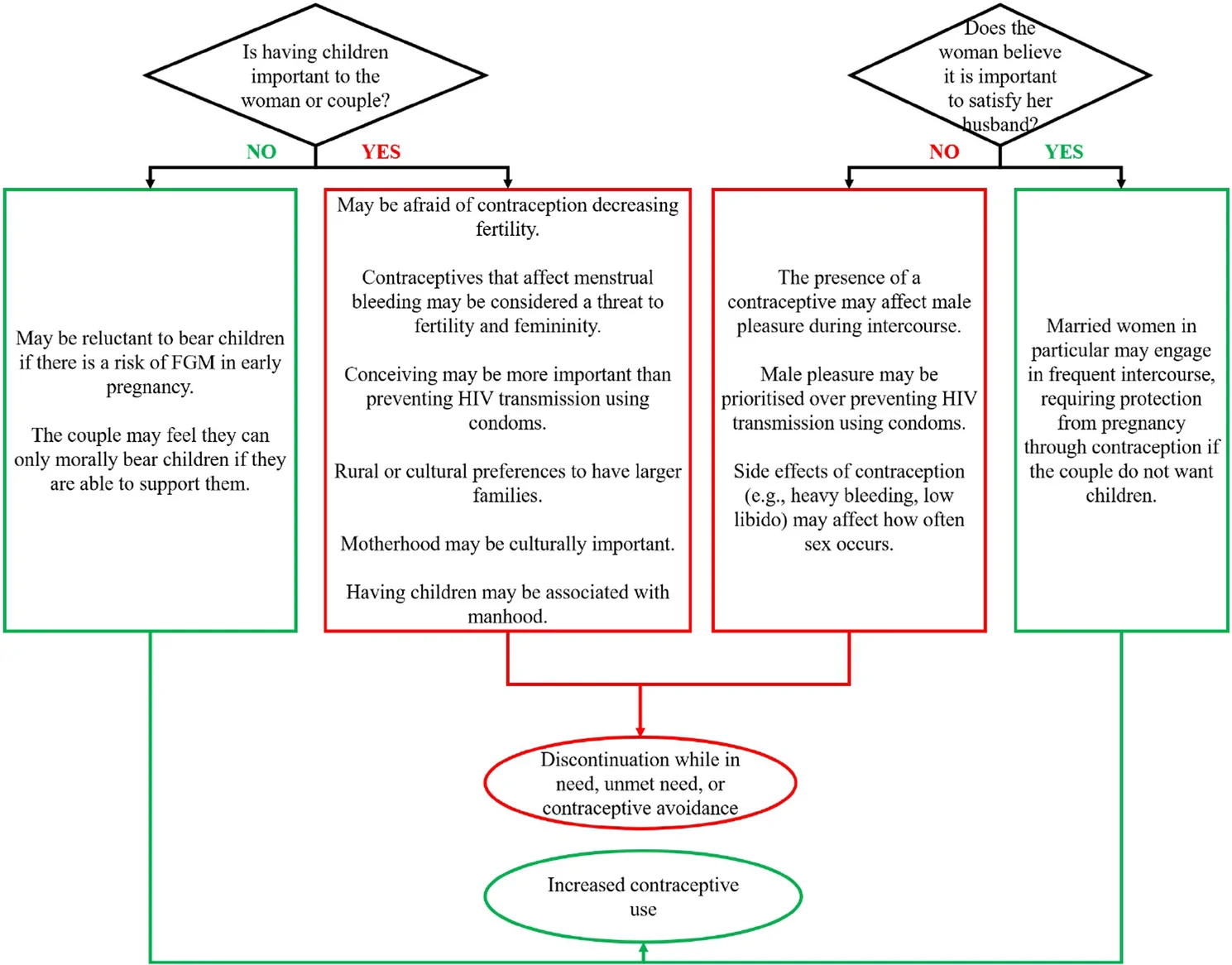
Impact of perceived marital duties on contraceptive use patterns

**Fig. 7:**
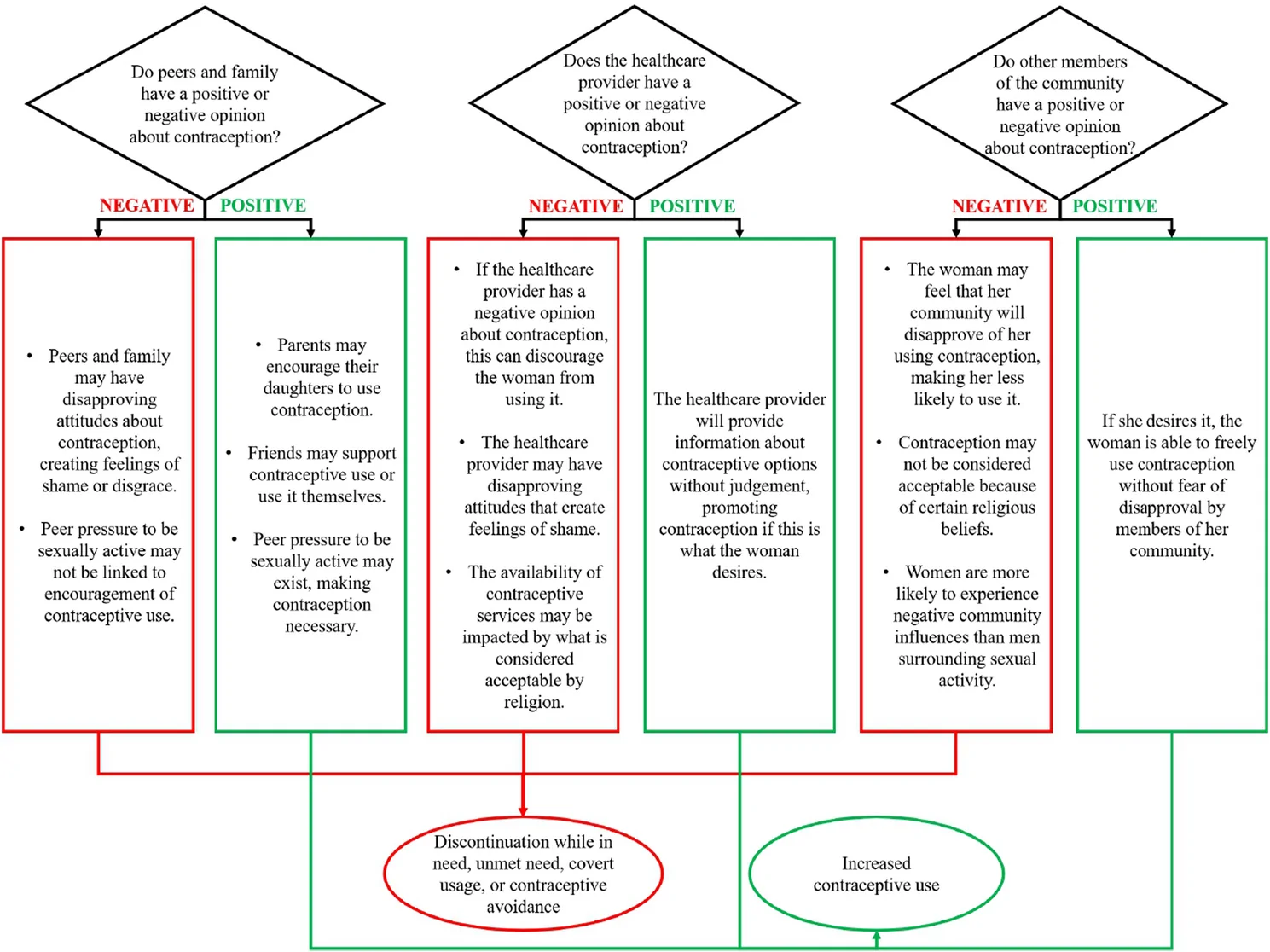
Impact of social network perceptions on patterns of contraceptive use

**Fig. 8:**
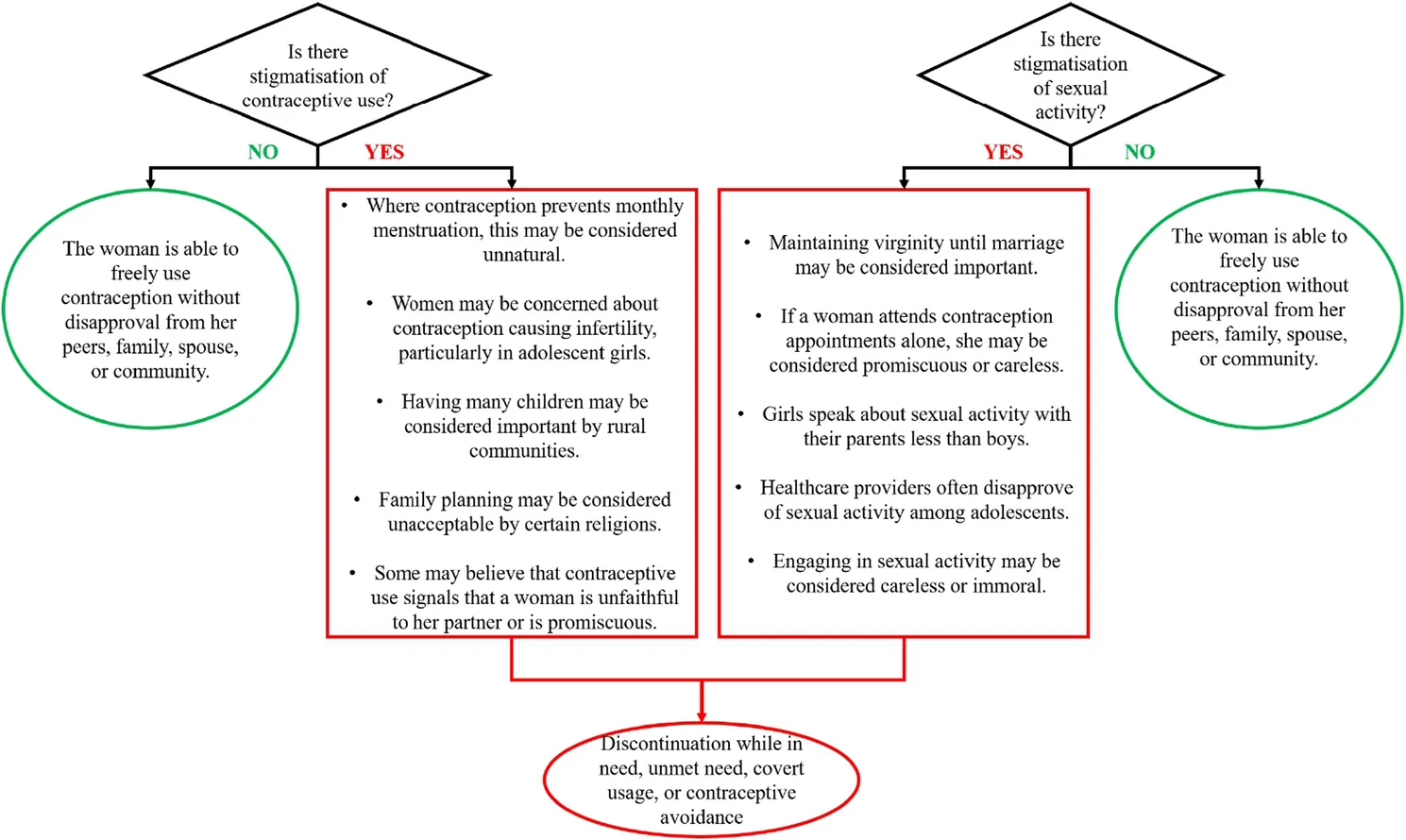
Impact of stigmatising beliefs regarding sexual activity and contraceptive use on usage patterns

**Fig. 9:**
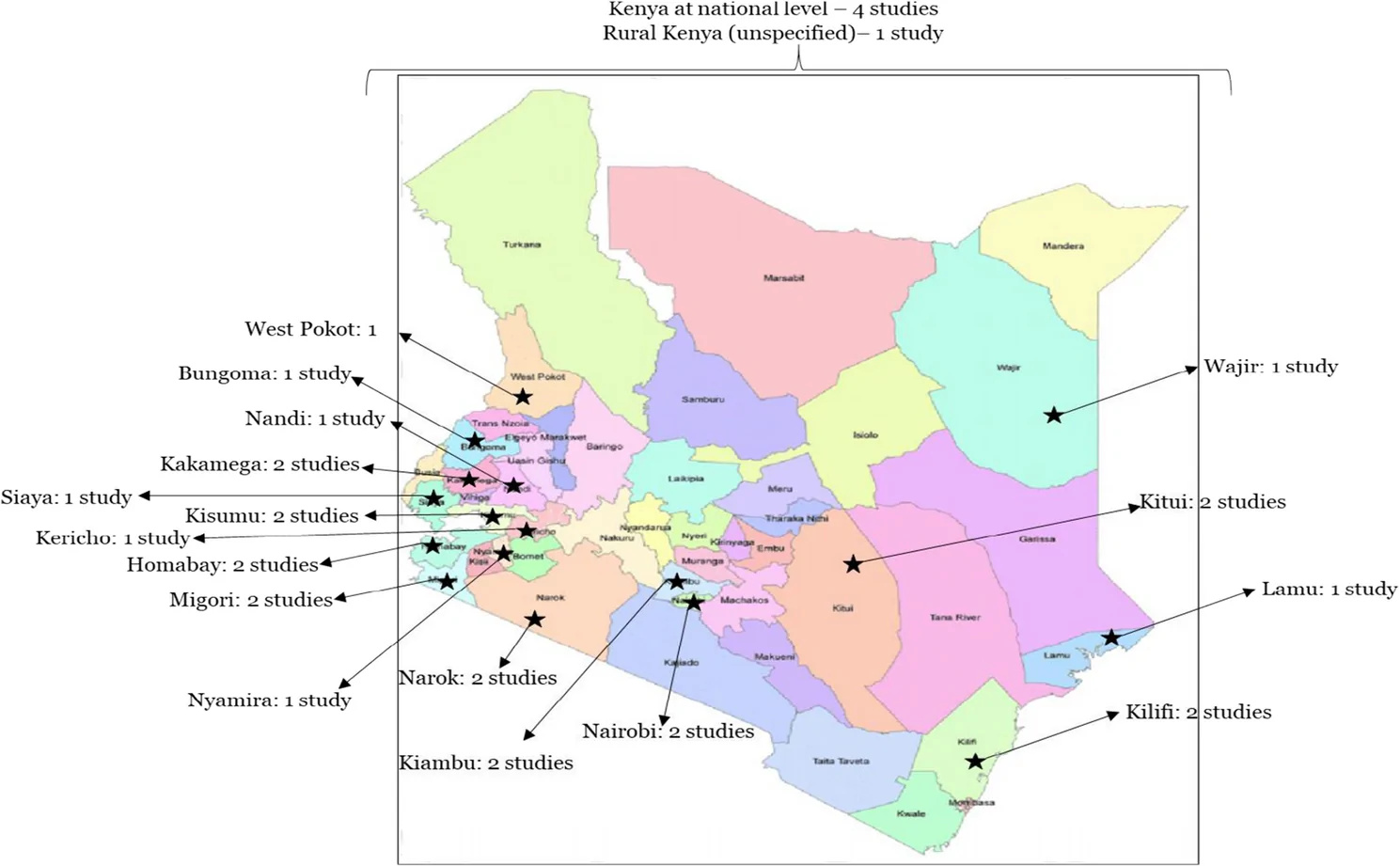
Geographical range of included studies, where highlighted regions indicate study locations represented in this review

Contextually, a broad range of populations were included, and data was reported at a variety of geographical levels, ranging from a village scope to the national level. Figures 3, 4, 5, 6, 7 and 8 summarise the key themes identified across the literature and their effect on contraceptive usage patterns, particularly whether phenomena falling under each theme serve as positive or negative predictors of usage. These themes consist of variations in female independence (as measured through education, employment and wealth), spousal effects, perceived marital duties of childbearing and provision of male pleasure, social network effects, and stigmatisation of sexual activity and/or contraceptive use. Finally, Fig. 9 visually represents the study locations represented in the literature, demonstrating that findings are largely clustered in western Kenya [16].

### Thematic analysis

The findings detailed below are most applicable to Western Kenya and therefore should not be considered representative of all regions of Kenya. Additionally, it bears noting that while not explicitly filtered for, all included studies only considered heterosexual relationships.

The gender-based influences on contraceptive use decisions can be interpreted by employing Kabeer’s conceptualisation of empowerment as constituted of material and human resources that shape the capacity for choice, decision-making agency that determines the ability to execute choice, and the achievement of contraceptive use where it is desired [11]. The observed patterns of contraceptive uptake, discontinuation while in need and unmet need may reflect the cumulative effect of gender on resource distribution and agency.

### Resources as a structural determinant of contraceptive decision-making

The first step of Kabeer’s framework is to identify the resources available to the target population group. Figure 3 demonstrates how material and human resources, namely a woman’s economic position, education and acquisition of knowledge, had a direct impact on her agency and decision-making/negotiation power within her relationship. Studies focused on the links between education and employment, negotiation power within intimate relationships, with informed knowledge surrounding different forms of contraception, the means to afford contraception without reliance on their marital partners, and practices of birth spacing [17, 18]. Where education, financial means and employment were present, these were positive predictors and enablers of contraceptive use, and the converse was indeed also true [43]. Figure 4 summarises the variation in education and employment status across the included studies where it was reported; there was a considerable range in employment (from just over 40% of participants to almost 70%), as well as in primary educational attainment (from under 40% to almost 80%), and in four of these studies, education and employment levels appeared to be correlated.

Poverty was a key barrier to contraceptive use. A study found that participants with limited socioeconomic means were 75% less likely to use contraception [19]. Injectable use in particular was associated with impoverishment [20], which may relate to the relative reduction in cost when using this method. In a study that showed that spousal consent and economic support from marital partners were required to access contraception [21], men were also considered to be ‘the owner of the pregnancy’. These phenomena reflect a tangible effect of material resources on contraceptive agency.

Studies demonstrated that education level, a human resource, was inextricably linked to informed contraceptive decision-making agency among women, and was inversely related to unmet need [17, 19, 20, 22–24]. This effect was pronounced; in Kakamega, tertiary-educated women were 2.58 times more likely to use long-acting contraceptives than those with primary education or below [22]. Likewise, in Wajir, secondary-educated women were 7.39 times more likely to use contraception [23]. Mechanisms for this phenomenon involve school-based education fostering an appreciation of the benefits of contraceptive use [24] and producing social mobility that allows new information about family planning to be acquired through interaction with others [17].

Education was also found to produce a social mobility characterised by increased engagement with radio, magazines and television [25], and interaction with a wider social network, leading to exposure to a wider range of views surrounding contraceptive use and sexuality [17]. The type of contraception chosen may also be influenced by education level. In one study, primary-educated participants were 54% less likely to use injectables as a covert method of exerting strategic agency where there is a fear of a partners’ disapproval, than those without primary education [19, 20]. Education was also considered an important prerequisite to obtaining employment and therefore securing financial independence [26] to afford contraception.

Acquisition of informational capital through contraceptive providers represented another important human resource. Levels of contraceptive knowledge and use were influenced by the quality of information provided by contraceptive providers [9, 21, 22, 24, 27]. Healthcare providers in Western Kenya, for example, were found to frequently and inappropriately restrict contraceptive counselling based on a patient’s parity, partner consent, and marital status, instead of clinically indicated criteria [28]. Contraceptive providers often did not adequately counsel users on side effects [22, 24], so knowledge was largely disseminated through potentially catastrophised anecdotes among social networks [22]. Nationally, just 51% of women received side effect information, and 81% sourced information from peers [9], with inaccurate anecdotal information inevitably discouraging contraceptive usage.

Additionally, where conversations surrounding female sexual activity and contraceptive use were avoided due to poor acceptability, community ‘shielding’ of knowledge occurred [24], such that in the absence of effective provider counselling, information was difficult to obtain. Interestingly, the studies revealed a gendered inequality in contraceptive knowledge. In one study, 75% of participant boys were familiar with contraception relative to 55% of girls [27].

### Agency shaped by relational decision-making

Studies showed that the opinions of heterosexual male partners heavily shaped contraceptive practices [43]. Figure 5 highlights that the presence of negative male opinions surrounding contraception, male objection to contraceptive use, and/or a relationship with a power disparity favouring the husband were all predictors of strategic agency in the form of covert usage, or a loss of agency that manifested as unmet need or discontinuation while in need. Male reproductive desires were often highly valued, such that alongside the fear of/actualised intimate partner violence where contraception was used despite spousal disapproval, they shaped contraceptive practices and usage.

Whereas some studies showed that just under half of women’s health decisions were made jointly between partners [18], others showed that gender-based power imbalances in heterosexual relationships were a key determinant of contraceptive use by women, with male partners frequently being the primary decision-maker on contraceptive decisions [25]. In Wajir and Lamu counties, for example, male disapproval was cited as a reason for contraceptive non-use by 78% of the sample, such that the incidence of contraceptive use was only 18.6% [23]. These findings highlight an enduring gender norm wherein male perspectives are prioritised over female ones, including in domains of women’s health.

In cases of HIV-serodiscordant relationships, where one partner had an HIV-positive status, male partners again dominated decision-making on when to use condoms [29]. Male partners’ strong expectations around fertility were a key barrier to contraceptive use, whereby family planning was only permissible in marriages that had already yielded children, and this often outweighed risks of HIV transmission and the desires of the female partner [29].

Where spouses exhibited extreme disapproval for contraceptive use, this was closely linked to fears of, or enacted, gender-based and intimate partner violence. This, in turn, led to a suppression of agency through coercive control (including contraceptive sabotage and pregnancy coercion) and possible restriction of resources that negatively impacted contraceptive decision-making, resulting in contraceptive discontinuation or avoidance [30]. For example, discovery of contraceptive use was linked to fears of/actualised beating [22] or physical removal of inserted contraceptive devices. Intimate partner violence victims may have been unable to obtain permission or funds, thus generating a threefold barrier to consistent contraceptive use [30]. There is a high incidence of gender-based violence in Kenya, with 41% of women having ever experienced economic, emotional, physical or sexual violence from their husband/intimate partner in 2022 [9]. This reinforces the pervasive gender norm of male dominance in relationships and is an important example of its profound impact on contraceptive behaviours.

In situations of transactional intercourse, power inequalities were found to impair the provider’s ability to self-advocate for contraceptive use and often prevented use [19], and hence served to suppress agency. Transactional intercourse also reflected a resource asymmetry, through economic dependency and a financial gradient between the sexual provider and client, which also reduced bargaining power and agency.

Similarly, in relationships where there were significant age differences, the power possessed by older male partners over adolescent girls prevented them advocating for safe sexual practice or partner support, leading to low attendance at contraceptive services [21, 24]. Furthermore, adolescent girls in poverty were disproportionately vulnerable to sexual exploitation compared to boys, where some girls traded sexual favours for money; this inherent dependency was not conducive to advocating for safer sex, including contraceptives [27]. These examples represent further manifestations of gender-based power imbalances that serve to negatively impact contraceptive usage.

Where male disapproval was a key influence in contraceptive decision-making, strategic agency was present, i.e., the covert use of contraceptive methods that were less detectable, and that were sufficiently long-acting so as not to require frequent access to contraceptive services [20, 24, 30]. Nationally, injectables were identified as the dominant method of choice, used by 50.4% of contraceptive users nationally [30]. In Wajir and Lamu counties, injectables were overwhelmingly prevalent, at 84% and 94% of the contraceptive-using sample respectively [23]. Injectables were overall considered the most effective method for facilitating covert use, as marital partners could detect implants through arm palpation and could feel intrauterine devices (IUDs) during intercourse [23]. It is possible that the pill may also have been a less favourable option due to its lower affordability (costing 63% more than the injectable per couple year of protection), alongside a higher failure rate due to skipped doses [31], and the potential risk of medication packets being found. These patterns highlight achieved reproductive outcomes that stem from constrained agency, instead of from free preference.

### Agency shaped by fertility & marital expectation

Heteronormative expectations of a woman’s ‘marital duties’ surrounding childbearing and provision of male pleasure may have served to discourage contraceptive usage, as summarised in Fig. 6. The mechanisms of this phenomenon included fears surrounding the effect of contraceptive use on fertility, the direct purpose of prevention of childbearing, and perceived interference of contraceptive use with male pleasure.

Childbearing duties were often a culturally important determinant of contraceptive non-use. In rural Kenya, women had more children and little unmet need [17, 23, 25, 29, 32]; childbearing was a logistically important role of rural women, as large families were required for fieldwork [25], limiting contraceptive need. In Wajir, a woman’s social standing was directly correlated with her number of children [23]. Similarly, in Luo culture, ‘manhood’ was achieved after bearing 10 children [32]. As aforementioned, the desire to conceive at times overshadowed the risk of HIV transmission between HIV-serodiscordant sexual partners [29]. Interestingly, unmet need was high in women with five or more children [17], which may have been due to continuing cultural expectations to bear children. For some, childbearing duties were considered religiously mandated, while others felt a moral obligation to limit their family size to ensure they could adequately provide for their children [25].

High value was placed on female fertility, therefore where contraceptive use was perceived as a threat to menstruation and fertility, usage was discouraged [21, 25, 33]. For example, 82.2% of participants in a vaginal ring study considered the absence of menstruation unnatural [33]. Fears surrounding difficulties becoming pregnant after discontinuation, or menstruation not returning, were a key factor reducing ring acceptability 25]. Similarly, 20% of healthcare providers in a study believed that contraceptive use would create persistent infertility [21], counselling adolescents towards abstinence as a result.

Contraceptive discontinuation or avoidance occurred where contraceptive use posed a threat to male pleasure [22, 29, 33], implying that its provision was considered a normative expectation of women. For example, contraceptives carrying side effects of reduced libido or menorrhagia (heavy menstrual bleeding) were found to impact sexual relationships [22], as was cessation/reduction in menstruation [22, 33]. Concerns about condom use interfering with sexual pleasure were also associated with avoidance of barrier contraception in HIV-serodiscordant relationships [29].

### Agency shaped by stigmatisation and social proof

The opinions of members within women’s social network were found to be key modulators of their contraceptive attitudes and usage behaviours. The studies revealed a culture of stigmatisation in Kenya that heavily influenced contraceptive use, as summarised in Fig. 8. Link & Phelan (2001) state that stigma occurs when individuals with a characteristic difference from others are linked to negative stereotypes by dominant cultural norms, separating them from others, and subsequently causing discrimination and status loss [34]. Behaviour is shaped by descriptive norms (what others do) and injunctive norms (what is considered permissible) [35]; therefore, stigma can be considered a sanction for norm violation, i.e., reproductive choices that deviate from what is expected or considered acceptable. In the studies identified, stigma related to themes of adolescent sexuality, premarital intercourse, or the perception of contraceptive usage as synonymous with promiscuity or infidelity.

Figure 7 demonstrates the influence of social network perceptions on contraceptive usage, illustrating the role of ‘social proof’ in shaping contraceptive behaviours. ‘Social proof’ refers to the process whereby individuals generate an understanding of which behaviours are appropriate based on the attitudes and behaviours of members of their social network [36]. Applied to contraceptive decision-making, women may interpret the attitudes, opinions, and behaviours of key referents within their social networks as indicators for which contraceptive practices are socially acceptable. Where key referents in a woman’s social network, such as peers, family, and healthcare providers, are opposed to contraceptive use and/or hold negative attitudes towards sexual activity or contraceptive use, this often translates to fear of or actualised stigmatisation that results in patterns of covert usage, unmet need, or discontinuation while in need, as summarised by Fig. 8. Negative attitudes towards contraceptive use may relate to it being perceived as unnatural, contrary to fertility ideals, posing a risk to fertility, or as indicating infidelity, with sexual activity itself potentially perceived as violating virginity expectations, or as evidencing carelessness, immorality and promiscuity [43].

Women were often therefore deterred from contraceptive use by its social consequences, which included strained interpersonal relationships, societal rejection and being labelled as ‘promiscuous’ or ‘unfaithful’ by spouses, healthcare providers, social networks and peripheral community influences such as religious leaders [9, 24, 27, 29].

Dual method contraceptive use in HIV is recommended to reduce transmission to unaffected partners (via the barrier method) and to prevent vertical transmission through pregnancy that might occur through human error in condom use(37). While recommended, selecting dual method usage, instead of sole barrier use or no contraceptive, was found to be frequently interpreted as evidence of infidelity among HIV-serodiscordant couples(29). The belief that contraception should be reserved for married women was reflected by higher rates of unmet need for unmarried women (19%) than married women (14%) nationally [9]. These findings may be reflective of a normative framing of non-use as a symbol of trust and sexual conservatism, with stigma arising as a consequence of norm violation.

Adolescents were particularly subject to stigmatisation [21, 27, 42]. Perceptions among healthcare providers included viewing the adolescent as having multiple relationships, not being serious about her relationship, or as being too young to engage in sexual activity without adopting a ‘careless’ attitude towards it [27]. As a result of these stigmatising beliefs, standoffish attitudes towards youths seeking contraceptive services were adopted by some providers [27], often driven by concerns that an adolescent’s contraceptive use would encourage promiscuity among her peers [21]. This serves to reinforce empirical expectations surrounding what is considered appropriate for an adolescent girl.

Similarly, some teachers perceived an adolescent using a contraceptive as “spoiled, immoral, lov[ing] men and [not concentrating on] studies”[21], even sending their pupils home after searching for or discovering contraceptive use. Returning to school with contraceptives also subjected girls to stigmatising peer attitudes [21]. Additionally, sexual activity was found to be perceived as less permissible among adolescent girls than boys [27], indicating a pronounced gender-based difference in views. Again, injectable contraceptives were deemed a preferred method within this population as a means of exerting strategic agency to evade stigmatisation, and knowledge about injectables and the pill was much higher than other methods [27]. Therefore, stigmatisation of sexual activity was found to be a likely mechanism of unmet need or covert usage among adolescent girls, reinforcing normative expectations of avoiding intercourse at this age.

This phenomenon operates differently to within marital relationships; in contrast to adolescent sexual activity being poorly accepted, childbearing (which necessitates sexual activity) often constitutes an important responsibility of the married woman, and a sign of virility for her spouse. However, the stigmatisation of adolescent sexual activity and the expectation of marital childbearing both point to the same outcome of low contraceptive acceptability.

## Discussion and conclusion

This scoping review defines the parameters of the gender-contraception nexus in Kenya, finding that (hetero)gendered norms interact with and influence contraceptive decision-making in a multifaceted manner and that contraceptive decision-making is a relational and socially embedded process. Across the included studies, women’s contraceptive choices were shaped by interactions between structural inequalities, interpersonal power relations, and community norms that collectively influenced both access to contraceptive resources and the ability to exercise reproductive agency. Kabeer’s conceptualisation of empowerment provides a useful framework for interpreting these findings, illustrating how resources, agency and achievements are mutually constituted. However, the review also suggests that these dimensions are themselves produced through broader gendered social relations.

The review suggests contraceptive decision-making practices are negotiated within relationships characterised by unequal distributions of power. Male heterosexual partners frequently occupied dominant decision-making positions regarding fertility and contraceptive use, with women’s reproductive preferences often subordinated to men’s expectations surrounding childbearing, sexuality and marital roles. Where women anticipated disapproval, coercion or violence, contraceptive use frequently became covert (particularly with the use of injectables) or was discontinued. These findings extend Kabeer’s framework by demonstrating that agency should not be understood simply as the capacity to make choices but as the ability to negotiate those choices within unequal social relationships.

The findings also demonstrate that contraceptive technologies carry important social meanings. Across the included studies, contraception was frequently associated with promiscuity, infidelity, infertility or rejection of expected gender roles. These meanings influenced decision-making independently of clinical concerns regarding efficacy or side effects and reflected broader moral understandings of femininity, sexuality and marriage; shaped by the social and cultural meanings attached to contraception within particular contexts.

Stigma emerged as a particularly important mechanism through which gender norms influenced contraceptive behaviour. Negative attitudes towards contraception among partners, families, peers, healthcare providers and religious leaders created social environments in which contraceptive use could threaten women’s social standing and intimate relationships. This was especially evident for adolescents, whose contraceptive use was frequently interpreted as evidence of sexual promiscuity or moral failure.

The review further highlights the role of healthcare providers. Providers frequently acted as gatekeepers whose counselling reflected prevailing social norms surrounding age, marital status, parity and sexuality. Restrictions on contraceptive access based on social norms, alongside inadequate counselling regarding side effects, contributed to misinformation. These findings suggest that healthcare institutions are themselves embedded within wider gendered systems of power and should not be viewed as neutral settings in which contraceptive decisions are made.

Taken together, these findings suggest that contraceptive practices provide an important lens through which broader systems of gendered power can be understood. Decisions surrounding fertility and contraception reflected wider inequalities in economic dependence, authority within intimate relationships, social respectability and expectations of womanhood. Although Kabeer’s empowerment framework effectively captures the importance of resources, agency and reproductive achievements, the review indicates that these dimensions are shaped by structural conditions operating across households, communities and institutions. In this respect, the WHO Social Determinants of Health framework offers a complementary perspective by locating women’s reproductive choices within wider structural determinants, including gender norms, education and socioeconomic position [41]. Rather than viewing these frameworks as competing explanations, the present findings suggest that they are mutually reinforcing; structural determinants influence the distribution of resources and opportunities for agency, while interpersonal interactions translate these structural inequalities into everyday reproductive decision-making.

### Limitations

While providing notable insight into gender norms influencing patterns of contraceptive use in Kenya, this scoping review is subject to methodological limitations. Firstly, some included studies also considered the effects of factors not related to gender (e.g., residence location or religion [23] which were not explored in this review, demonstrating that the gender norms-contraceptive usage association is multifactorial and subject to confounding associations. Therefore, while this review uncovers an important contribution of gender to contraceptive use and decision-making, the findings ought to be considered within broader social determinants. Secondly, scoping was confined to a single platform; while a detailed keyword search within a comprehensive platform aimed to mitigate omissions, articles outside this were not considered, and grey literature was not searched; the inclusion of grey literature and additional search platforms could be a valuable avenue for furthering the presented research. However, as themes repeated across the academic literature, the review likely tended towards data saturation. Though keywords were generated based on variables elicited from preliminary research, it is possible that some relevant concepts were not identified and included in the search. Creation of the a priori protocol, while allowing space for later expansion of search terms, therefore did not fully mitigate against the risk of unintentional exclusion of ideas during the scoping process. The search filters employed meant that insights from non-English articles were excluded in the interest of avoiding translation error.

Additionally, while included papers focused on a variety of locations and demographics, as mentioned, Western Kenya was over-represented in the included studies (Fig. 9), with many central regions being omitted entirely, reducing the generalisability of findings at the country level. Also, though not filtered for, the included studies only discussed heterosexual relationships. Results extracted from included studies were also subject to limitations of the source study’s methodological design, such as the ability to establish temporal precedence and causality, response rate or sample size concerns, recall or social desirability bias, and poor generalisability of the results of qualitative studies. Similarly, the nature of scoping review reporting means that findings are conveyed using the same sociological lens as their source study. During critical appraisal, assigning a 0.5-point value to ‘Unclear’ items on checklists may have caused a marginal overstatement of the quality of studies where reporting of the quality item was unclear. Moreover, the generation of eligibility criteria and search terms, alongside the screening and data extraction, were conducted by the first author due to resource constraints, which deviates from PRISMA-ScR recommendations for dual independent screening and introduces a small possibility of human error and bias. However, a sensitivity check of a subset of excluded studies did not identify discrepancies in the application of eligibility criteria, and themes were consistent across the literature, assuring their identification while charting.

### Avenues for intervention and further research

These findings highlight potential interventional opportunities for improving contraceptive use. Table 4 summarises possible avenues for intervention based on the findings uncovered in this review, alongside the mechanism by which they are likely to improve contraceptive uptake, and supported by evidence of efficacy from Kenya, with consideration of possible limitations of each approach.

**Table 4:** Avenues for Intervention

Intervention	Associated Finding	Mechanism of Action	Evidence from Kenya	Limitations
Promoting female access to economic resources, through vocational training, employment opportunities, and financial independence programmes.	Enhancing female agency through education, employment, and acquisition of wealth, facilitates contraceptive use.	This may enhance female empowerment by increasing material and human resources, and subsequently reproductive agency to enable contraceptive choice.	Vocational training centres in Nakuru Town improved access to employment opportunities and wealth for women and, by extension, their decision-making autonomy [38].	Negative perceptions of community vocational training may impede enrolment and course completion [38].
Male engagement initiatives designed to promote male support for contraceptive use.	Widespread male-dominated decision-making patterns exist in relationships. Male opinions may involve stigmatising concepts.	Involving men in contraceptive discourse might serve to positively influence their opinions surrounding contraception and begin to dispel stigmatising concepts, such as contraceptive use being indicative of promiscuity or infidelity. Male support for contraceptive use may lead to increase uptake by female partners.	The AQCESS programme in rural Kenya provided targeted information delivery to men using male authority figures and joint counselling of couples, which successfully improved male knowledge of, and support for, reproductive health [39].	It is important to avoid excluding women from contraceptive discourse. Male involvement must be encouraged using a gender-transformative approach, to avoid reinforcing scenarios where male dominance has negative consequences on female agency or safety. Strong attitudes towards contraceptive use may be difficult to influence.
Integration of HIV, contraceptive and domestic violence services to improve accessibility of contraceptive and domestic violence services.	There is a need for unbiased contraceptive education and discourse among HIV serodiscordant couples. Extreme spousal disapproval for contraceptive use may be associated with intimate partner violence. Stigmatisation of contraceptive use and sexual activity may occur alongside fears of the medical impacts of contraceptive use.	Adequately training providers to offer unbiased contraceptive counselling could occur through provision of a culturally competent and sensitive course designed following in-depth discussion with service users about their experiences, concerns, and reproductive care priorities. This would be valuable in tackling stigmatisation of sexual activity and contraceptive use, including by dispelling misconceptions about the impact of contraceptive use on infertility, and addressing perceived associations with promiscuity or infidelity. Additionally, attendance at an integrated service can support women who can only use contraception covertly, as it offers a degree of anonymity regarding which service is being accessed. The ability to concurrently access domestic violence services during the contraceptive appointment can also be valuable in protecting women against intimate partner violence that may be associated with extreme spousal disapproval.	Offering integrated HIV-family planning services at high-volume facilities has been found to increase contraceptive uptake and reduced unmet need, supporting routine integration as a means of increasing contraceptive use [40].	Offering integrated HIV-family planning services at predominantly low-resource facilities with a high volume of patients may be logistically and financially difficult.

Across the interventions suggested, there are three recurring implementation barriers that should be considered when designing interventions. Firstly, challenges are likely to arise when implementing services in resource-constrained settings; integrated services in particular require staffing, training, equipment, and funding, limiting the scalability of such interventions. Secondly, entrenched sociocultural norms, beliefs, and stigma may be difficult to reverse and may affect engagement with new programmes. Finally, implementation must also remain highly gender-sensitive to avoid reinforcing male authority and/or inadvertently diminishing women’s agency. Taken together, these challenges suggest that interventions may be most effective when supported by culturally sensitive provider training, community-based advocacy, and integration within existing healthcare and community infrastructures, thereby promoting smoother uptake and sustainability. Further research should explore how gender-based sociocultural norms vary by demographic group or geographic location to facilitate targeted, resonating interventions, and consider the prevailing gender norms in regions that are under-represented by the existing literature.

## Supplementary Information

Supplementary Material 1.

Supplementary Material 2.

Supplementary Material 3.

Supplementary Material 4.

## Data Availability

The datasets generated and/or analysed during the current study are available from the corresponding author on reasonable request.

## Abbreviations

DHS: Demographic and health survey
HIV: Human Immunodeficiency Virus
IUD: Intrauterine device
JBI: Joanna Briggs Institute
PCC: Population, Concept, Context
PRISMA-ScR: Preferred reporting items for systematic review and meta-analysis protocols (Scoping reviews)
SDH: Social Determinants of Health
