# Supplementary material for: Gender and Contraception in Kenya: a Scoping Review
Source: Reprod Health. 2026 Sep 24;23:177. doi: 10.1186/s12978-026-02443-5 (PMC13625416; doi:10.1186/s12978-026-02443-5)
Supplement: Supplementary file 1 [file 12978_2026_2443_MOESM1_ESM.docx]

**Appendices**

**AdditionaFile 1** Concept map of keywords to inform search *(created via Canva Whiteboard)*.
