# Supplementary material for: Gender and Contraception in Kenya: a Scoping Review
Source: Reprod Health. 2026 Sep 24;23:177. doi: 10.1186/s12978-026-02443-5 (PMC13625416; doi:10.1186/s12978-026-02443-5)
Supplement: Supplementary file 2 [file 12978_2026_2443_MOESM2_ESM.docx]

**Additional File 2** Keywords inputted into ProQuest.

impact* OR influence* OR effect* OR consequence* OR implication* OR determinant*

AND

perception* OR perceive OR perceived OR aware OR awareness OR knowledge OR value* OR principle* OR belief* OR view* OR viewpoint* OR impression* OR moral* OR expectation* OR feeling* OR duty OR duties OR societ* OR societal OR culture* OR cultural OR attitude* OR opinion* OR thought* OR misconception* or misinformation* OR information* OR misunderstanding* OR misinterpretation*

AND

gender norm* OR gender role* OR expectation* OR relationship*

AND

education OR educated OR school OR schooling OR employment OR employed OR career OR work OR working OR childbearing OR pregnancy OR birth OR carry children or carrying children OR carrying a child OR carry a child OR reproduction OR reproductive or autonomy OR authority OR submission OR submissive OR submissiveness OR submission OR equality OR inequality OR chastity or virginity OR premarital sex OR premarital sexual intercourse OR premarital intercourse OR premarital sexual activity OR sexual activity OR sex OR pleasure or honor Or honour OR stigma or stigmatisation OR stigmatised or rights OR empowerment OR modern OR traditional

AND

contraceptive* OR birth control OR contraception OR family planning OR pregnancy prevention OR birth prevention OR condom* OR diaphragm* OR barrier* OR pill OR implant OR coil OR IUD OR intrauterine device* OR intra-uterine device* OR IUS OR intra-uterine system* OR intra-uterine system* OR injection* OR depo OR patch* OR vaginal ring* OR intravaginal ring* OR female sterilisation OR tubal ligation

AND

behaviour OR abstinence or abstain OR avoidance OR avoid OR covert OR hidden OR hide OR concealed OR conceal OR secret OR stealth OR stealthy OR inconsistent OR intermittent OR irregular OR sporadic OR improper OR continuous OR continued OR uninterrupted OR long term OR long-term OR short term OR short-term OR regular OR use OR take OR consume OR usage OR utilisation OR administration

AND

woman OR women OR female* OR girl* OR adolescent* OR husband* OR partner* OR boyfriend* OR spouse* OR intimate partner* OR man OR men OR male* OR famil* OR relative* OR elder* OR household head* OR mother* OR father* OR parent* OR brother* OR sister* OR sibling* OR household* OR communit* OR generation OR extended famil*

AND

childbearing age OR fertile OR fertility OR childbearing potential

AND

Kenya OR Republic of Kenya or Kenyan Republic

(Limit to: Full text, Peer reviewed, publication after 1/1/2014, English, Scholarly Journals, Location Kenya NOT all other non-Kenyan regions).
