# Supplementary material for: Gender and Contraception in Kenya: a Scoping Review
Source: Reprod Health. 2026 Sep 24;23:177. doi: 10.1186/s12978-026-02443-5 (PMC13625416; doi:10.1186/s12978-026-02443-5)
Supplement: Supplementary file 3 [file 12978_2026_2443_MOESM4_ESM.docx]

**Additional File 4** Characteristics and critical appraisal scores of included papers, with both fractional (Unclear = 0.5) and binary (Unclear = 0) items presented.

| **Authors and Year** | **Region** | **Population and sample size** | **Study design** | **Focus on gender norms & contraceptive use** | **Critical appraisal score & justifications** |
| --- | --- | --- | --- | --- | --- |
| Abdi et al. (2024). | Wajir and Lamu – predominantly rural. | Muslim women, aged 18-49.  350 women sampled in each county (700 total). | Analytical cross-sectional study. | Patterns and determinants of contraceptive use in these two predominantly Muslim settings. | 6/8 items (75%) – *confounding variables, or strategies to deal with them, were not identified.*  *Binary scoring:6/8 (75%)* |
| Hagey et al. (2015). | Homabay, Kisumu & Migori – combination of urban and rural areas. | Healthcare providers in hospitals and dispensaries, which had the highest HIV prevalences in Kenya, and provided contraceptive services.  40 healthcare providers sampled. | Cross-sectional, observational, mixed methods study with a strong qualitative component. | Barriers and facilitators of contraceptive access among adolescents living with HIV. | 8.5/10 items (85%) – *researcher not located culturally or theoretically, impact of author-participant relationships on author responses to study events is unclear.*  *Binary scoring: 8/10 (80%).* |
| Hakansson et al. (2018). | Kisumu East District – mixed urban and rural. | Various healthcare providers experienced in post-abortion care and youth-friendly services.  86 post-abortion care providers sampled. | Nested convergent mixed-methods sub-study within a cluster randomized controlled trial. | Stigmatising attitudes among healthcare providers surrounding adolescent pregnancy, abortion and contraceptive use. | 8.5/10 items (85%) – *researcher not located culturally or theoretically, impact of author-participant relationships on author responses to study events is unclear.*  *Binary scoring: 8/10 (80%).* |
| Kirui et al. (2021). | Kilifi, Nairobi, Kiambu, Nyamira, Nandi, Bungoma, Kericho, Siaya, Kitui, Kakamega & West Pokot – predominantly rural, with some urban areas. | Women of reproductive age who were using a modern contraceptive method at the time of the study.  2450 women sampled. | Analytical cross-sectional study. | Factors associated with an increased uptake of injectable contraceptives among users of modern contraceptive methods. | 6/8 items (75%) – *confounding variables, or strategies to deal with them, were not identified.*  *Binary scoring: 6/8 (75%).* |
| Lahiri et al. (2023). | 2 villages in rural Kilifi county. | Male and female adolescents who were married, living with a partner, or unmarried and not cohabiting.  763 respondents sampled across both villages. | Cross-sectional social network (sociocentric) study. | Influence of individual and group-level normative influence on modern contraceptive use. | 6.5/8 items (81.25%) – *study stated that confounding variables were controlled for, but what the confounders were, and what strategies were implemented, not stated.*  *Binary scoring: 6/8 (75%).* |
| MacQuarrie & Mallick (2021). | Kenya at the national level. | Married women using modern, temporary contraception for 12 months before the interview.  1437 married women sampled. | Cross-sectional analytical study. | Impact of intimate partner violence on contraceptive discontinuation while still in need. | 8/10 items (80%) – *researcher not located culturally or theoretically, and influence of researcher on research (and vice-versa) not addressed.*  *Binary scoring: 8/10 (80%).* |
| Manguro et al. (2021). | Narok – mixed urban and rural. | - Adolescent girls aged 15-19 who were ever pregnant, or never pregnant. - Boys aged 15-19. - Informants: parents of adolescent girls, community leaders, health service providers & teachers.   114 ever pregnant girls, 123 never pregnant girls, and 63 boys sampled. | Cross-sectional mixed-methods study. | Drivers for sexual activity, access to sexual and reproductive health services, and barriers to contraceptive use. | 7/8 items (93%) *– strategies to deal with confounding factors were not stated.*  *Binary scoring: 7/8 (93%).* |
| Mankelkl et al. (2024). | Kenya at the national level. | Women of reproductive age, from both rural and urban areas, who used contraception.  14,987 women sampled. | Cross-sectional study. | Factors associated with modern contraceptive utilisation. | 6/8 items (75%) – *confounding variables, or strategies to deal with them, were not identified.*  *Binary scoring – 6/8 (75%).* |
| Musalia (2017). | Kenya at the national level. | Married women of both urban and rural residence, and of varying socioeconomic status.  4931 married women sampled. | Qualitative study. | Decision-making profiles among Kenyan women, and the predictive nature of socioeconomic variables on household decision-making power distribution. | 8/10 items (80%) – *researcher not located culturally or theoretically, and influence of researcher on research (and vice-versa) not addressed.*  *Binary scoring – 8/10 (80%).* |
| Nyauchi & Omedi (2014) | Rural Kenya (unspecified). | Women of reproductive age, who were not pregnant or amenorrhoeic ,and belonged to a range of socioeconomic backgrounds.  5829 women sampled. | Cross-sectional analytical study. | Factors contributing to high unmet need for family planning among rural Kenyan women, and their importance in child-spacing and birth limitation choices. | 6/8 items (75%) - *confounding variables, or strategies to deal with them, were not identified.*  *Binary scoring – 6/8 items (75%).* |
| Okoli et al. (2019). | Kenya at the national level. | HIV-positive women of reproductive age.  304 HIV-positive women sampled. | Cross-sectional analytical study. | Association between sociodemographic factors and contraceptive use. | 8/8 items (100%) – *no quality deficits.*  *Binary scoring – 8/8 (100%).* |
| Ontiri et al. (2019). | Kakamega County – predominantly rural. | - Women of reproductive age who visited family planning clinics to start contraception. - Healthcare providers of contraceptive methods.   423 women sampled. | Cross-sectional study. | Sociodemographic and reproductive factors associated with long-acting reversible contraceptive uptake. | 8.5/10 items (85%) – *researcher not located culturally or theoretically, impact of author-participant relationships on author responses to study events is unclear.*  *Binary scoring – 8/10 (85%).* |
| Ontiri et al. (2021). | Kitui & Migori – rural counties. | Adolescent mothers and other women of reproductive age, who discontinued a modern contraceptive, with or without their spouses present.  135 participants sampled. | Cross-sectional qualitative study. | Experiences with previous contraceptive use, reasons for discontinuation and future intention to use, in women with unmet need for contraception. | 6/8 items (75%) – *confounding variables, or strategies to deal with them, were not identified.*  *Binary scoring – 6/8 (75%).* |
| Ormel et al. (2021). | Narok and Homabay – mixed urban and rural. | - Young people (aged 18-24). - Community health volunteers. - Informants – community and youth leaders, NGO and government programme staff.   37 interviews and 15 focus group discussions (participant number unknown). | Qualitative study. | Factors influencing the role of community health volunteers in access and uptake of contraceptive services by youths. | 9/10 items (90%) – *no statement locating researcher culturally or theoretically.*  *Binary scoring – 9/10 (90%).* |
| Roxby et al. (2016). | Nairobi – urban. | HIV-serodiscordant couples who had regular intercourse, where the seropositive partner had not started ART and did not have advanced HIV.  34 interviews, with couples interviewed separately (participant number unknown). | Qualitative study. | Barriers to, and motivations for, dual contraceptive use in Kenyan HIV-serodiscordant couples. | 8.5/10 items (85%) – *researcher not located culturally or theoretically, impact of author-participant relationships on author responses to study events is unclear.*  *Binary scoring: 8/10 (80%).* |
| Walker et al. (2023). | Thika, Kiambu – mixed urban and rural. | Women aged 18-40 who:   - Met Amsel’s criteria for BV. - Were likely to have HIV or a partner with HIV. - Used a vaginal contraceptive ring to prevent pregnancy.   45 women sampled. | Randomised controlled trial with an embedded observational sub-study. | Influences on attitudes towards menstrual suppression among those using a vaginal contraceptive ring. | 9/11 items (82%) – *unclear whether participants were lost to follow-up and how this was dealt with. Unclear whether confounding factors were identified, and how they were dealt with.*  *Binary scoring: 7/11 (64%) – falls below 75% threshold.* |

The 75% critical appraisal threshold is considered to be an appropriate choice, as evidenced by the following sensitivity analysis. If the threshold were to be increased to 80%, 5 studies would have fallen below this value based on their fractional scores, and 7studies based on their binary scores;. While employing a quality threshold was not used to exclude studies from the analysis, the 80% threshold would have somewhat attenuated the meaningful contributions of the 5-7 studies not meeting it. Conversely, if the threshold were lowered to 70%, one study would have fallen below this value based on its binary score, while all fractional scores would have remained above the threshold, resulting in limited differentiation of quality.
